# Allogenic Amniotic Tissue for Treatment of Knee and Hip Osteoarthritis

**DOI:** 10.3390/ph15040404

**Published:** 2022-03-26

**Authors:** Ashim Gupta

**Affiliations:** 1 Future Biologics, Lawrenceville, GA 30043, USA; ashim6786@gmail.com; 2 BioIntegrate, Lawrenceville, GA 30043, USA; 3 South Texas Orthopaedic Research Institute (STORI Inc.), Laredo, TX 78045, USA; 4 Veterans in Pain (V.I.P.), Valencia, CA 91354, USA; 5 General Therapeutics, Cleveland Heights, OH 44118, USA

**Keywords:** osteoarthritis, knee osteoarthritis, hip osteoarthritis, regenerative medicine, biologics, amniotic tissue, amniotic fluid, amniotic membrane, amniotic suspension, amniotic-fluid-derived stem cells

## Abstract

Osteoarthritis (OA) impacts millions of people and places a high burden on healthcare systems in the United States. Current treatment modalities have limitations and do not address underlying pathology. Lately, there has been an immense growth in the use of biologics, including perinatal allogenic tissues for orthopedic regenerative medicine applications. Amniotic tissue is an exciting new alternative for such applications. Despite several published studies that reported its use for treatment of ophthalmic conditions and complex wounds, there are limited clinical studies evaluating its safety and efficacy in treating patients suffering with knee or hip OA. In this manuscript, I focused on three prospective clinical studies which evaluated the safety and efficacy of amniotic tissue in patients suffering with moderate knee or hip OA. The results from these studies presented the scientific community with much needed, well-executed, and prospective clinical trials. Though these trials demonstrated that administration of amniotic tissue in knee or hip joint is safe and potentially effective, more multi-center, prospective, double-blinded, randomized controlled trials are warranted to further establish the efficacy of amniotic tissue to mitigate symptoms of knee and hip OA to ultimately justify its clinical use.

Osteoarthritis (OA) is the most widespread joint ailment in the United States, impacting over 30 million adults, and this number is expected to reach 67 million by 2030 [1,2]. Its pathophysiology is associated with inflammation and decline in vascularization in the degeneration of articular cartilage [1]. This leads to substantial pain and reduced function [1]. OA normally affects larger weight-bearing joints, including hips and knees [1]. Conventionally, OA is managed with activity modification, immobilization, physical therapy, pharmacological agents, and surgical interventions after conservative therapies have been unsuccessful [3]. These treatment modalities have shortcomings, regularly trying to reduce pain rather than focusing on underlying pathology [4].

Over the previous decade, a few molecular targets, such as interleukin-1, transforming growth factor-β, matrix metalloproteinases, etc., have been discerned as mediators of OA [5,6,7]. Though some of these targets are encouraging, they may generate treatments with high risk-to-benefit ratio [8,9]. Therefore, alternative safe and effective treatment modalities are needed to address this unmet medical necessity.

Recently, there has been a remarkable growth in use of biologics for regenerative medicine applications, particularly in the field of orthopedics [10]. Biologics presently used in clinical practice include platelet-rich plasma, lipoaspirate, bone marrow concentrate, and perinatal allogenic tissue [11]. Perinatal allogenic tissue includes amniotic tissue (amniotic membrane and amniotic fluid), and its uses have advanced for treatment of different medical disorders such as ophthalmic conditions and complex wounds [12]. Though increasingly popular, there are limited high-level, peer-reviewed clinical studies demonstrating the safety and efficacy of amniotic tissue for treatment of patients suffering with knee or hip OA.

In this manuscript, we focused on three prospective studies, two for knee OA and one for hip OA. These studies are carried out by Vines et al. [13], titled, “Cryopreserved Amniotic Suspension for the Treatment of Knee Osteoarthritis”; Farr et al. [14], titled, “A Randomized Controlled Single-Blind Study Demonstrating Superiority of Amniotic Suspension Allograft Injection Over Hyaluronic Acid and Saline Control for Modification of Knee Osteoarthritis Symptoms”; and Meadows et al. [15], titled, “A Single Injection of Amniotic Suspension Allograft Is Safe and Effective for Treatment of Mild to Moderate Hip Osteoarthritis: A Prospective Study”. These studies are an effort from the authors of these manuscripts to build on published peer-reviewed preclinical studies [16,17,18,19,20] that analyzed the effect of amniotic tissue on OA. Briefly, these studies are discussed below:

- Willet et al. [16] utilized Lewis rat OA model with medial meniscus transection (MMT) followed by randomizing treatment groups to receive either saline or micronized dehydrated human amniotic/chorionic membrane (μ-dHACM) injections. In addition, a group of rats that did not undergo MMT received similar injections of saline or μ-dHACM. The results showed that the surgically treated rats that received μ-dHACM had a significant reduction in cartilage damage, including fewer focal defects and less attenuation, as compared to controls.
- Raines et al. [17] also utilized a Lewis rat OA model with MMT, and injections of saline or human cryopreserved particulate amniotic membrane/umbilical cord (AM/UC) at 50 μg/mL or 100 μg/mL doses were administered. The results showed that at 1 week post-injection, both AM/UC groups had a significant reduction in lesion area compared to the control group. Moreover, the rats that received the high-dose AM/UC injection showed augmented cartilage thickness and volume at 1 week and a significant decrease in lesion size at 4 weeks compared to the low-dose AM/UC and saline groups. Lastly, rats that were injected with AM/UC had significantly higher Osteoarthritis Research Society International (OARSI) histologic joint scores compared to the controls.
- Marino-Martinez et al. [18] utilized a rabbit model and induced OA in bilateral knees and administered human lyophilized AM in one knee and saline into the contralateral knee. The results demonstrated reduced cartilage damage at 3 and 6 weeks post-injection in the treatment knees compared to the control knees.
- Reece et al. [19] utilized a rat model with MMT and injected μ-dHACM comprising two different particle sizes, with saline injection as a control. The results showed that standard μ-dHACM led to diminished cartilage degeneration, but decreased particle size μ-dHACM resulted in heightened roughness of cartilage.
- Kimmerling et al. [20] designed a chemically induced knee OA model in rats and treated with saline, triamcinolone, or amniotic suspension allograft (ASA) in 25 μL or 50 μL doses. On behavioral assays, they reported significant improvements in pain threshold with reduced weight-bearing aversion and swelling in the ASA-treated rats, though no differences in histological grading scores were observed.

In the first study by Vines et al. [13], the authors performed a prospective, open-label study to evaluate feasibility of an intraarticular injection of amniotic tissue for knee OA (Kellgren–Lawrence grade 3 or 4 tibiofemoral knee OA) and gathered preliminary data on safety and efficacy. Six patients were enrolled in the study, administered a single intraarticular amniotic tissue injection, and followed for a period of 12 months. No major adverse events were reported related to injection of amniotic tissue, and all the safety data was within the reference range of the laboratory during the course of the study. Patient-reported outcome measures, including International Knee Documentation Committee (IKDC), Knee Injury and Osteoarthritis Outcome Score (KOOS), and Single Assessment Numeric Evaluation (SANE), were collected during the course of study and assessed for up to 12 months of follow-up visit. Due to small sample size, statistical analysis was not performed. In addition, absence of a control group impeded any analysis in terms of efficacy. Nevertheless, improvements in scores for IKDC, KOOS and its subscales, and SANE were observed. Even though this study has limitations, the authors should be commended for their efforts as they were one of the first groups to perform a clinical study that demonstrated use of amniotic tissue for treatment of knee OA.

Attributing to the data from the pilot study [13], demonstrating safety and trends for improved pain and function, a second study by Farr et al. [14], a multicenter randomized controlled trial, was performed to determine the efficacy of amniotic tissue compared to hyaluronic acid (HA) and saline in patients with moderate knee OA (Kellgren–Lawrence grade 2 or 3). A total of 200 patients were randomized 1:1:1 to amniotic tissue, HA, and saline groups and were blinded to their allocation. Patients received one of the three injections, based on their allocated group, and were followed for a period of 6 months. The results from this study reported better outcomes for amniotic tissue compared to HA and saline. Specifically, significant differences were observed between amniotic tissue and HA group at 3-month follow-up for EQ-5D-5L pain and anxiety subsets, KOOS pain, activity of daily living (ADL) and symptoms subscales, and VAS score for overall pain, and pain through strenuous work and normal daily living. Significant differences were also observed for KOOS symptoms subscale at 3 months between amniotic tissue and saline groups. At 6-month follow-up, amniotic tissue showed better improvement compared to both HA and saline group for EQ-5D-5L pain, activities, mobility and health today subscales; KOOS pain, ADL and symptoms subscales; SANE; and overall pain on VAS. A significantly higher responder rate for amniotic tissue compared to HA and saline group was also reported based on the OMERACT-OARSI (Outcome Measures in Arthritis Clinical Trials—Osteoarthritis Research Society International) set of responder criteria. One limitation of this study would be that it ended up being a single-blinded study instead of a double-blinded study. However, it will be difficult to make it a double-blinded study as investigators can differentiate between the injectables used, due to differences in their inherent viscosities. Nonetheless, as most of the end-points were patient reported, it reduced the bias from unblinded investigators. Another limitation of this study was brief duration of follow-up, i.e., 6 months. To overcome this, Gomoll et al. [21] published another follow-up study at 12 months, titled, “Safety and Efficacy of an Amniotic Suspension Allograft Injection Over 12 Months in a Single-Blinded, Randomized Controlled Trial for Symptomatic Osteoarthritis of the Knee”. The results from this follow-up study reported no concerning immunologic or adverse events, and significant improvements in VAS and KOOS scores that were maintained up to 12 month of follow-up. One of the overall limitations of these studies [14,21] is the high dropout rate, attributed to unacceptable pain levels at end of 3 months, especially for patients treated with placebo. In spite of this, these studies are the first ever systematic randomized controlled blinded studies evaluating safety and efficacy of a perinatal allogenic tissue in patients suffering from knee OA. I applaud the efforts of the authors and hope to see larger, possibly phase III, randomized controlled trials along with some basic science studies demonstrating mechanism of action of amniotic tissue in ameliorating knee OA.

In the third study, by Meadows et al. [15], the authors evaluated the effect of amniotic tissue in patients suffering with moderate (Tonnis grade 1 or 2) hip OA. Ten patients were enrolled in the study, received a single image-guided injection in the hip joint, and followed for a period of 12 months. No major adverse events were reported during the course of the study. One patient failed treatment, and data were analyzed for the remaining nine patients. A significant improvement was reported at 12 months compared to baseline for International Hip Outcome Tool Scores. A significant improvement was also reported for SANE scores at 6 months compared to baseline and at 12 months compared to baseline. Similar outcome was reported for Modified Harris Hip Scores both at 6 months and 12 months compared to baseline. No evidence of deteriorating joint space narrowing was found during the course of this study. Notably, the mean improvement in the patient-reported outcome measures exceeded minimal clinical improvement difference (MCID), implying a considerable clinical improvement. This study has few limitations, including small sample size and absence of a control group. Despite this, to my knowledge, this is the first clinical study evaluating the efficacy of amniotic tissue in mitigating symptoms associated with mild to moderate hip OA.

These studies are not without shortcomings. The authors of these articles described amniotic tissue as amniotic suspension allograft that contains human amniotic membrane and human amniotic fluid-derived cells. No protocol for formulation of this allograft along with its composition was described. Another shortcoming is availability of limited information related to mechanism of action of amniotic tissue to combat OA. Few basic science studies, including one from Kimmerling et al. [20], demonstrated that post-injection, there is an increase in the levels of interleukin-10 and induction of monocytes to become M2 instead of M1. They both help reduce the effects of inflammatory cytokines, decrease levels of proinflammatory cytokines, and increase expression levels of anti-inflammatory cytokines. Though the basic science studies for utilization of amniotic tissue are limited, studies involving other similar biologics, such as PTP-001 (amnion-chorion and umbilical cord product), reported that administration of this product in an inflammatory model led to reduction in expression levels of tumor necrosis factor and interleukin-1β. It also showed anticatabolic effect via reduction of matrix metalloproteinase-13 expression levels [22]. Similarly, a study involving umbilical-cord-derived Wharton’s jelly showed presence of several growth factors, cytokines including anti-inflammatory cytokines, hyaluronic acid, and extracellular vesicles, including exosomes [23]. The authors concluded that presence of multiple factors within one formulation may help reduce inflammation, decrease pain, and augment healing of musculoskeletal injuries, including OA [23,24]. Another study involving cell-free stem-cell-derived extract formulation demonstrated presence of growth factors, cytokines including anti-inflammatory cytokines, and exosomes [25]. This study also showed increased rate of cell proliferation and stem cell migration post-treatment with this formulation [25]. The authors concluded that presence of multiple factors, including exosomes within one formulation, along with the ability to promote cell proliferation and induce stem cell migration may reduce inflammation and pain, and augment tissue repair [25,26]. Based on these basic science preliminary studies, I believe that presence of right constituents (e.g., growth factors, cytokines, exosomes, etc.) in these formulations is likely responsible for their ability to potentially treat OA.

In summary, despite these limitations and inadequate knowledge of mechanism of action, the authors of these articles [13,14,15,21] presented the scientific community with much-needed, well-executed, and promising prospective clinical trials. These trials, in my view, definitely demonstrated that administration of amniotic tissue in knee or hip joints is safe and they also laid the foundation for essential, prospective, larger, double-blinded, randomized controlled trials to further establish the efficacy of amniotic tissue to mitigate symptoms of knee and hip OA, thereby ultimately justifying its clinical use. In addition, more basic science studies determining the mechanism of action of amniotic tissue in mitigating OA are needed. Moreover, studies comparing efficacy of amniotic tissue to gold standard treatments such as corticosteroids, platelet-rich plasma, etc., are warranted to further our understanding and define its efficacy profile more accurately.

As of 1 March 2022, there are no ongoing clinical trials registered on ClinicalTrials.gov for hip OA related to amniotic tissue. For knee OA, there are five ongoing studies listed related to amniotic tissue, which are summarized in Table 1.

## Figures and Tables

**Table 1:** Clinical trials registered on ClinicalTrials.gov till 1 March 2022 utilizing amniotic tissue for treatment of knee osteoarthritis.

Study Identifier	Tissue Type	Study Phase; Estimated Enrollment (N)	Primary Outcome Measure(s)	Recruitment Status	Country
NCT04612023	Acellular Amniotic Membrane	Phase 2; N = 90	(1) Primary Efficacy Endpoints using Validated patient-reported outcome tools questionnaires (timeframe: 1 year)—Knee injury and Osteoarthritis Outcome Score (KOOS)—assess five outcomes: pain, symptoms, activities of daily living, sport and recreation function, and knee-related quality of life. It is a 0–100 scale, with zero representing extreme knee problems and 100 representing no knee problems. (2) Primary Efficacy Endpoints using Validated patient-reported outcome tools questionnaires (timeframe: 1 year)—Western Ontario and McMaster Universities Osteoarthritis Index (WOMAC)—assess the condition of patients with osteoarthritis of the knee and hip, including pain, stiffness, and physical functioning of the joints. It is a 0 (worst) –96 (best scale. (3) Primary Efficacy Endpoints using Validated patient-reported outcome tools questionnaires (timeframe: 1 year)—Visual Analogue Scale (VAS)-assess pain, It is a 0–100 scale. A higher score indicates greater pain intensity.	Recruiting	USA
NCT04636229	Amniotic suspension (Amniotic membrane + amniotic fluid-derived cells)	Phase 3; N = 474	(1) The difference in change from baseline in WOMAC pain scale at 6 months between ASA- and placebo-treated patients (timeframe: baseline to week 26)—The Western Ontario and McMaster Universities (WOMAC^®^) Osteoarthritis Index is a questionnaire that measures pain, stiffness, and function both independently and collectively, using a Likert 3.1, 5-point scale. The Likert Scale uses the following descriptors for all items: none, mild moderate, severe, and extreme, corresponding to an ordinal scale of 0–4. Higher scores on the WOMAC indicate worse pain, stiffness, and functional limitations.	Recruiting	USA
NCT03441607	Micronized human amnion chorion membrane	Phase 2; N = 320	(1) Visual Analogue Scale (VAS) (timeframe: 3 months)—Decreased Pain Level.	Unknown	USA
NCT04886960	Amniotic Fluid	Phase 1/2; N = 60	(1) Repeat allogeneic intra-articular injection within 6 months (timeframe: 6 months)—Participants in both the SOC and pAF treatment arms may require and/or request rescue medication (i.e., SOC injection) at any time and will be given per PI discretion as part of standard of care. The clinicians will not know which study arm the study participant is in but will treat the participant with the SOC injection. This information will be documented and collected in the Electronic Medical Record (EMR), as well as the study’s electronic data capture system. Participants will not be given any additional pAF injections throughout the study period. The participant will continue to be treated with SOC injections as needed. The outcome will be an indicator of whether or not a subject received a rescue medication within 6 months.	Recruiting	USA
NCT04698265	Amniotic suspension (Amniotic membrane + amniotic fluid-derived cells)	Not applicable; N = 150	(1) Change of the Western Ontario and McMaster Universities Arthritis Index (WOMAC) between baseline, 1 week, and 1, 3, 6, 12 months (timeframe: baseline, 1 week, 1, 3, 6, 12 months)—WOMAC is a self-administered questionnaire consisting of 24 items divided into three subscales: (1) Pain (5 items): during walking, using stairs, in bed, sitting or lying, and standing upright (2) Stiffness (2 items): after first waking and later in the day(3) Physical Function (17 items): using stairs, rising from sitting, standing, bending, walking, getting in/out of a car, shopping, putting on/taking off socks, rising from bed, lying in bed, getting in/out of bath, sitting, getting on/off toilet, heavy domestic duties, light domestic duties. The test questions are scored on a scale of 0–4, which correspond to None (0), Mild (1), Moderate (2), Severe (3), and Extreme (4).	Not yet recruiting	Taiwan

## Data Availability

Not applicable.
